# Six-year analysis of posterior zirconia crowns with vertical margin preparations

**DOI:** 10.1038/s41415-024-7951-1

**Published:** 2025-05-23

**Authors:** Jane Stack, Brian Millar

**Affiliations:** 530665951180147069556 General Dental Practitioner, Exeter Advanced Dentistry, United Kingdom; 639305362209750705095 Clinical Professor of Dental Education, Consultant in Restorative Dentistry, Faculty of Dentistry, Oral and Craniofacial Sciences, King´s College London, United Kingdom

## Abstract

**Background** The application of vertical margins for crown preparations is increasing in popularity but limited long-term clinical outcome data exist.

**Aim** To audit clinical outcomes for 73 teeth after vertical preparation for knife-edge zirconia crowns on posterior teeth in general practice.

**Methods** In total, 73 posterior teeth had knife-edge zirconia crowns placed after vertical finish line preparation. The outcomes for these teeth were analysed from a retrospective audit of clinical records.

**Results** The mean follow-up time was 62 months (SD: 21 months; range: 12-93). Eight teeth experienced complications during the follow-up period, with two of these teeth ultimately being extracted (one due to tooth fracture and one due to caries at the crown margin). The remaining six teeth were still functioning. Three of these had crown replacements, one required re-root canal treatment and two teeth (in the same patient) had unstable periodontitis which worsened since placement of crowns. Only the patient with unstable periodontitis showed any change in alveolar bone height on radiographic follow-up; 68 teeth with radiographic follow-up available showed no changes in alveolar bone levels. The mean bleeding score for the crowned teeth was higher than the mean bleeding score for the control tooth, but this was not statistically significant.

**Conclusion** This retrospective evaluation has shown favourable outcomes for 71 teeth after vertical preparation for knife-edge crowns. Longer follow-up is needed but the present results show that the technique is a viable procedure with potential advantages.

## Key points

- This paper describes a new technique to crown teeth with minimal preparation.
- Highly successful outcomes are documented.
- The benefits of this aesthetic technique are described.

## Introduction

Crowns are frequently used to restore the function and appearance of broken-down teeth. In the United Kingdom, the Adult Oral Health Survey 2021 reported that 34% of those surveyed self-reported to have at least one crowned tooth.^1^ This compares closely to the results from the 2009 survey, where a physical examination was carried out and 37% of adults were found to have at least one crown, increasing to 59% in patients over 45 years.^2^ Of the adults with crowns, each had an average of three crowns.

The application of adhesive dentistry has enabled alternative restorative techniques to be used in some clinical situations, which may mean that crowns are prescribed less frequently than in the past, but they still have relevance for heavily restored teeth and crown replacements. The most common reasons for the replacement of an existing crown are aesthetics (21%), recurrent caries (20%), lost crown (15%) and crown fracture (15%).^3^

When providing a crown, the clinician needs to decide on the margin type and position. This will be governed by material used, aesthetics and existing tooth damage and restorations. If an existing crown is to be replaced, then it may be necessary to prepare a new finish line more apically than the previous finish line. This makes it more likely that the crown will finish on the root surface, making conservation of tooth structure even more important.

The depth of the crown margin preparation will influence the amount of coronal tooth structure removed. The percentage of crown removal during preparation has been shown to vary between 56% for a full gold crown to 75.6% for a full metal ceramic crown.^4^ A pulpal inflammatory response is more likely if more dentine is removed.^5^ This is especially relevant in the cervical region where there is less dentine thickness to begin with. Zirconia is growing in popularity and provides an aesthetic crown which is strong in a thin section, with a conventional chamfer margin (0.3-0.5 mm) commonly used.^6^

If a crown preparation is necessary, it is essential to provide the least invasive preparation. A knife-edge crown on a vertical margin requires less tooth reduction of the axial wall, particularly in the cervical region.

Pardo classified finish lines as horizontal or vertical.^7^ A horizontal preparation has a clearly defined finish line, such as a chamfer or shoulder, which allows space for the restorative material and communicates to the laboratory technician the precise location of the desired crown margin. A vertical preparation does not have a defined finish line and includes teeth prepared with a knife-edge or feather-edge and the advantages and disadvantages of these types of margin have been described previously.^8^

One of the original indications for vertical margins was for crowning periodontally involved teeth. In these cases, the use of a horizontal margin would necessitate excessive root surface reduction, as well as excessive removal of coronal tissue. Di Febo illustrates the use of different margins, including feathered margins, chamfers and shoulders in the ‘combined preparation technique' at the level of the bony crest in order to even out concavities associated with root furcations.^9^ Later, the 20-year follow-up report was published.^10^

Vertical margins result in crowns with very thin marginal areas and therefore require a material that is very strong and resistant to fracture in a thin section. This has been possible for decades, with metal margins, such as those in full gold crowns and porcelain fused to metal crown with a metal-only collar. More recently, zirconia has been developed as a crown material and can also be fabricated with a thin margin. This zirconia option allows excellent aesthetics and conservation of tooth structure with a vertical preparation when restoring severely damaged teeth.

In vitro studies have shown improved marginal fit of crowns with knife-edge margins over shoulder margins and this can also be explained mathematically.^11^ There is a decreased space between the tooth preparation and the crown due to vertical geometry. As the marginal angle reduces from a shoulder of 90^0^ (often used for ceramic materials) through chamfers of 45^0^ and 30^0^ (often preferred for metal and zirconia) to a vertical margin of 0^0^, the marginal gap also reduces. Thus, in theory, a crown with a vertical margin preparation has a marginal gap as close to zero as possible.

The use of a vertical preparation with an incorrect emergence profile (overcontour) could lead to a crown margin overhang with associated plaque retention and gingival inflammation. Careful preparation design and soft tissue handling from the clinician are essential. A skilled technician, familiar with this technique, is key to achieving the correct emergence profile and finishing to avoid gingival inflammation.

There are different methods for vertical preparation. The biologically oriented preparation technique (BOPT) was the original vertical preparation technique and aims to create a replacement crown with an improved emergence profile.^12^^,^^13^^,^^14^ During crown preparation, there is gingitage of the internal wall of the gingival sulcus and a provisional restoration is worn for a 4-8-week healing period to allow for gingival remodelling before definitive impressions are taken. The alternative ‘shoulderless approach', as applied in the current analysis, requires placement of the final restoration as soon as possible so that soft tissue regeneration is guided by the contours of the definitive restoration.^15^ These techniques (and other variations, including featheredge preparations and vertical margin closure) have been described and compared.^8^

As there is a lack of clinical studies reporting on the use of vertical margins, the aim of this audit was to evaluate the clinical outcomes for 73 teeth prepared with a vertical finish line preparation and restored with a zirconia knife-edge crown, placed in general practice. This is a longer-term follow-up to our previously published audit.^8^

## Methods

Data collection was carried out in 2024 by analysing patient records in a private practice in England. All patients who had been treated for one or more posterior single crowns over a three-year period (2016-2018) were identified. Only patients who were treated with a vertical margin crown preparation and had follow-up data were included in this audit. This follow-up data were recorded during routine examination and hygiene appointments. The most recent examination was used as the follow-up date. The mean follow-up time was 62 months, with a standard deviation of 21 months and a range of 12-93 months.

A total of 73 knife-edge zirconia crowns were included. These preparations were carried out by two separate clinicians following the same protocol. The follow-up data were recorded by clinicians and hygienists at the practice as part of ongoing dental care. Crown survival, marginal integrity, endodontic health and periodontal health were checked at subsequent examination appointments and these data were audited.

Each individual tooth received a vertical shoulderless preparation created with a non-cutting tip bur and an occlusal reduction of approximately 2 mm. The technique has been described in detail previously.^8^ In each case, a provisional crown was made chairside from a bisacryl composite (Luxatemp; DMG, Germany) using a pre-operative putty matrix. This provisional crown margin was then adapted with a flowable composite to ensure marginal integrity and adequate emergence profile.

Zirconia knife-edge margin crowns were fabricated using computer-aided design/computer-aided manufacturing (CAD/CAM). Some crowns were entirely monolithic zirconia, while those in a more visible position were veneered with porcelain to improve aesthetics.

**Fig. 1:**
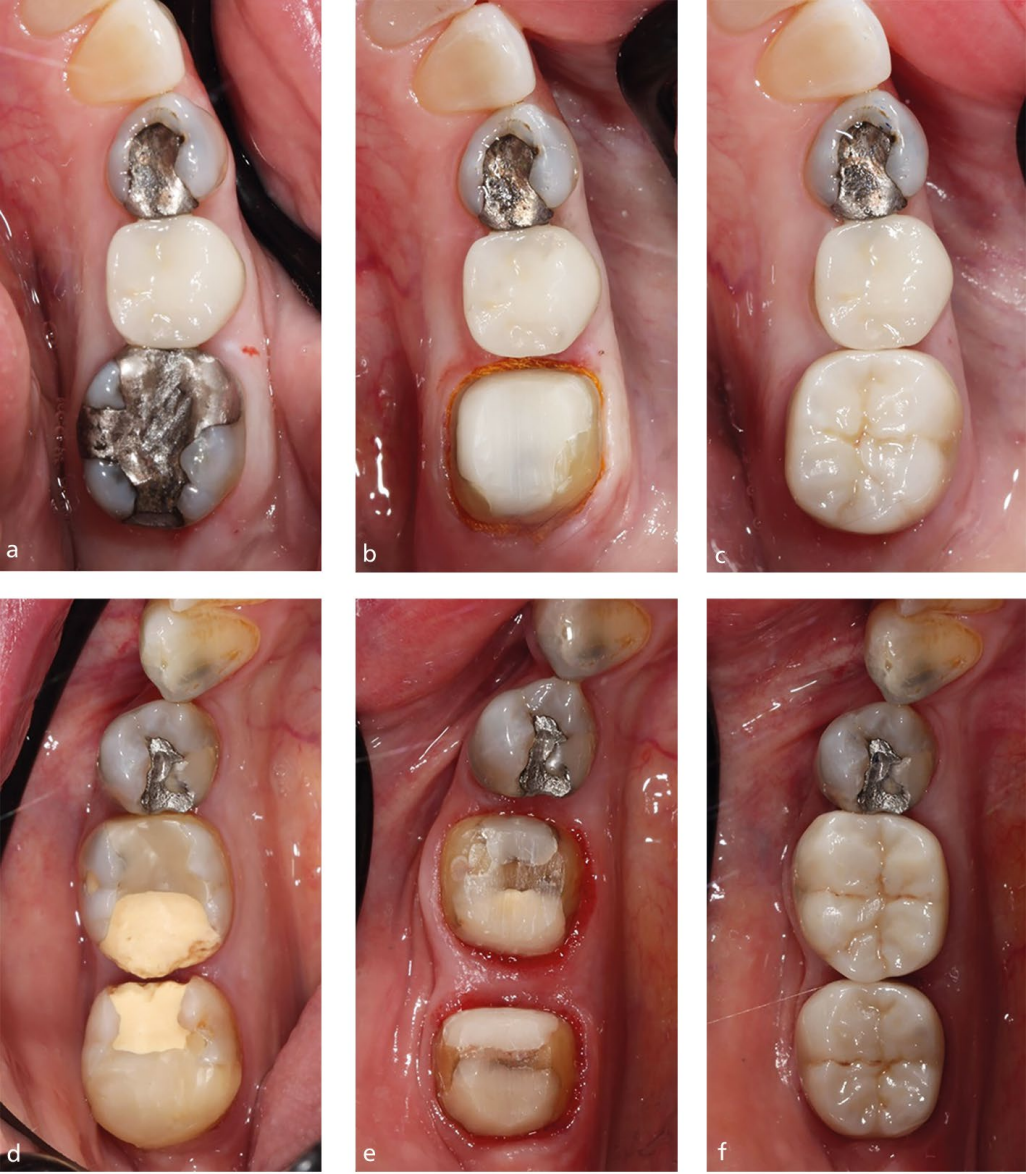
a, b, c, d, e, f) Two clinical cases showing pre-op, tooth preparation and crown fit

Crowns were cemented after approximately two weeks with Rely-X Unicem or Ketac Cem (3M ESPE; Seefeld, Germany). An example of the technique is shown in Figure 1 and has been published in detail previously.^8^

Patients re-attended for examination and hygiene appointments based on their individual risk profile, which was most commonly every six months. Basic periodontal examination (BPE) scores were recorded at each visit and periodontal pocketing was recorded for any patients who recorded a BPE score of 3 or 4. Bleeding charts were recorded when needed. Bleeding charts were available for 69 of the teeth in the audit. The data were calculated as number of surfaces with bleeding on probing. If there was bleeding on mesial and palatal surfaces, this was counted as two surfaces. If there was more than one bleeding record available, an average was calculated.

Bite-wing radiographs were taken for patients to aid caries diagnosis, at an interval appropriate to their caries risk profile. Crowns were visually evaluated at each examination for any chips, cracks or marginal gaps.

A control tooth was chosen for comparison. This was another crowned tooth in the mouth. Contralateral teeth were first choice, followed by opposing or adjacent crowns. Where no other crowns were available, a heavily filled contralateral tooth was chosen. For the following comparisons, teeth prepared for a vertical preparation crown will be referred to as ‘crowned teeth' while the control teeth chosen for comparison will be referred to as ‘control teeth'.

## Results

In total, 73 crowns had follow-up records which could be evaluated for this audit. Of these 73 crowns, 30 were placed by clinician one, and 43 were placed by clinician two. Both clinicians followed the same protocol and outcomes were broadly similar, with both experiencing complications (detailed below).

Table 1 shows the distribution of crowns according to tooth. In total, 45 were replacement crowns and 28 were new crowns. The reasons for crown placement included secondary caries, a lost or fractured existing crown, restoration of a root-treated tooth, a cracked tooth, or replacement of a crown with poor margins. In total, 62 of the teeth had the impression recorded at the same visit as preparation. The mean follow-up time was 62 months, with a standard deviation of 21 months and a range of 12-93 months.

**Table 1:** Distribution of crowns by tooth type

Distribution of crowns	Number
Maxillary premolars	13
Maxillary molars	31
Mandibular premolars	5
Mandibular molars	24
Total	73

There were complications with eight teeth during the audit period. Six teeth had restorative or endodontic complications, with two of these teeth ultimately extracted. Two other teeth (in the same patient) had increased periodontal pocketing and bone loss in a patient with unstable periodontitis.

One tooth fracture occurred during this audit period. The tooth in question (17) was the last remaining maxillary molar; all other maxillary molars had been extracted due to deep caries and associated complications. This tooth was deemed restorable, prepared for a crown with milled rest seats, and was then clasped by a cobalt-chrome removable partial denture. After 12 months, the tooth fractured at cervical level and needed to be extracted.

Three crowns needed to be re-made during the audit period. One crown had to be remade at the fit stage as the buccal wall fractured during try-in. Two crowns had to be remade at later stages, one after 77 months and one after 62 months. In both cases, the underlying tooth was sound, and a new crown was fabricated and fitted.

A total of 37 teeth were root-treated before crown preparation. This included recent root treatments and longstanding root treatments. Also, 36 of the teeth had no previous root treatment. One root-treated tooth required re-root canal treatment after 35 months. This was completed through an access cavity in the crown, which was then sealed with composite resin. This crown is still present and functional. None of the teeth which were not previously root-treated required subsequent root canal treatment during the audit period.

One tooth was found to have caries at the margin during the audit period. This was detected during a routine examination appointment after 56 months and there were no associated symptoms. The patient was offered further restoration to keep this tooth but instead chose to have it extracted. These complications are summarised in Table 2.

**Table 2:** Complications associated with crowned teeth during the audit period

	Tooth	Type of failure/complication	Time elapsed since crown preparation	Treatment provided	Progress of tooth since complication?	Clinician
1	36	Crown fracture at margin	Two weeks (day of fit appt)	Remake crown	No issues (61 months follow-up)	1
2	17	Tooth fracture at cervical level	12 months	Extraction	N/A	1
3	36	Tooth needed root canal treatment to be re-done	35 months	Re-RCT through access cavity in crown, sealed with composite resin	No issues (49 months follow-up after RCT)	2
4	27	Caries at crown margin, no symptoms	56 months	Extraction	N/A	1
5	17	Crown fracture, tooth underneath sound	62 months	Crown replacement	No issues (32 months follow-up)	2
6	15	Crown fracture, tooth underneath sound	77 months	Crown replacement	No issues (7 months follow-up)	2

Radiographic follow-up was available for 69 teeth. In most cases, this was bite-wing radiographs, but a few cases had before and after peri-apical radiographs to compare. Radiographs were only taken when clinically necessary. The mean follow-up time for radiographs was 55 months with a standard deviation of 19 months. The range was 1-87 months. No change in alveolar bone level was noted when comparing 67 of these teeth before and after placement of a crown with vertical margins. Two teeth (26 and 27 in the same patient with unstable periodontitis, discussed below) showed an increase in interproximal bone loss on bite-wing radiographs taken after 69 months.

Of the 73 teeth in this audit, 70 had follow-up BPE scores. Periodontal pocket charting was recorded for teeth found to have pockets greater than 3.5 mm.

Of these 70 teeth, seven teeth (in six patients) had a periodontal pocket recorded either before or after treatment with the vertical preparation (Table 3). Two of these teeth (crowns three and four from Table 3) were the same patient. In this patient, periodontal pocketing has significantly worsened in the 78 months since crown placement. The 26 shows an increase from 6 mm to 7 mm distal pocketing and the 27 shows an increase from 1 mm to 8 mm distal pocketing. This patient was the only case with visible bone loss evident on radiographs. Two other cases showed an increase in periodontal pocketing (to 4 mm) and three other cases, who had pocketing before crown placement, had no pocketing in subsequent records. All of the above patients underwent periodontal maintenance therapy with a hygienist, and all periodontitis is stable, with the exception of the patient mentioned above (crowns three and four).

**Table 3:** Details on teeth with associated periodontal pocketing (Case 3 and 4 are in the same patient)

	Tooth number (FDI)	Periodontal pockets before crown preparation?	Periodontal pockets after crown preparation?	Change in pocket depth	Bone loss seen on radiograph?
1	16	None recorded, BPE 3 for this sextant	4 mm mesial pocket	Possible increase, patient is a smoker and has other pockets around molars	No
2	36	None recorded	4 mm buccal pocket at one exam, no pockets at seven other exams; contralateral control tooth has Grade 1 furcation	Increase at one exam, otherwise stability	No
3	26	6 mm distal pocket	7 mm distal pocket	Increase	Yes
4	27	1 mm distal pocket	8 mm distal pocket	Increase	Yes
5	26	5 mm distal pocket	No pockets	Decrease	No
6	36	4 mm lingual pocket	No pockets	Decrease	No
7	37	5 mm mesial pocket	No pockets	Decrease	No

**Fig. 2:**
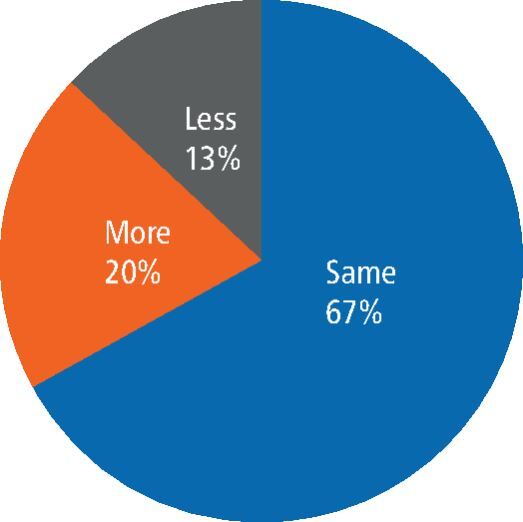
Graph showing comparison of bleeding on the crowned tooth and a control tooth

The bleeding score was calculated for each tooth from records taken before and after vertical preparation for a crown. This was compared with a control tooth, where bleeding was found to be more, less, or the same as the control tooth. Figure 2 represents this comparison. Of the 69 teeth with bleeding data available, 14 showed more bleeding on the crowned tooth, nine showed less bleeding, while 46 teeth had the same level on the crowned tooth and control tooth.

The mean bleeding score for the crowned teeth (0.519) was higher than the mean bleeding score for the control teeth (0.417). A two-sample t-test (SPSS Version 29) was used to test the null hypothesis that the means for the crowned teeth and the control teeth were equal versus the alternative hypothesis that the means were not equal (see Table 4). The assumptions underlying the two-sample t-test were satisfied. The 69 crowned teeth (mean bleeding score = 0.519; standard deviation = 0.889) were compared with 69 control teeth (mean bleeding score = 0.417; standard deviation = 0.695). The 95% confidence interval for the difference in means (crowned teeth versus control teeth) was -0.167, 0.37. The hypothesis test did not show a significant result (p = 0.457), so the null hypothesis was not rejected. In conclusion, the mean bleeding score for the crowned teeth and the control teeth are not different.

**Table 4:** Bleeding on probing - comparing the crowned teeth with the control teeth

	Bleeding on probing (number of surfaces) on crowned teeth	Bleeding on probing (number of surfaces) on control teeth
Number (N)	69	69
Mean	0.519	0.417
Standard deviation	0.889	0.695
Median	0	0
Range	0-4	0-2

## Discussion

This audit reports positive outcomes for posterior teeth prepared with a vertical preparations for zirconia crowns placed in general practice. The mean follow-up time was 62 months, with a standard deviation of 21 months and a range of 12-93 months. The outcomes are similar to those previously reported.^8^

There were two failures requiring extraction of the tooth, both in patients with a high caries rate and a history of multiple extractions, as previously reported.^8^ There were three crown failures requiring a refabrication of the crown. The first occurred at a crown fit appointment; the buccal margin fractured during try-in. On this occasion, the thin-section zirconia was not strong enough to resist fracture. This crown was remade and has been functioning well for the past 61 months. Two more crowns fractured later, one after 77 months and one after 62 months. In both cases, the underlying tooth structure was sound and the crowns were remade.

None of the teeth without root treatment prepared with vertical preparations required subsequent endodontic treatment, possibly due to the reduced depth of axial preparation required. One previously root-treated tooth required re-treatment after 35 months, which was carried out through the crown, and the crown is still functioning well 49 months later. Ongoing review will enable comparison with others who report that 20% of vital teeth prepared for metal-ceramic crowns require endodontic treatment within 15 years.^16^ These recorded results compare favourably with the data published by Poggio *et al*.^17^ on 102 knife-edge zirconia crowns and by Schmitt *et al*.^18^ on 19 featheredge zirconia crowns.

Two teeth showed an increase in periodontal pocketing; these were both in the same patient with unstable periodontitis. Before treatment, this patient had periodontitis and poor crown margins on the 26 and 27. These crowns were replaced with zirconia knife-edge crowns on vertical preparations and have been reviewed over the past 78 months. For approximately five years, the periodontitis was broadly stable, but in the past year, pockets have increased and bone loss has been evident on radiographs.

Bleeding data were compared for the crowned tooth and a control tooth, which was usually a crown with a traditional margin, or a heavily filled tooth. This allowed some comparison with horizontal margins; although, it is recognised that there are many other factors at play. The crowned teeth were found to have a higher mean bleeding score than the control teeth, but this difference was not significant. These results compare favourably with data published by Scutella *et al*. on the periodontal assessment of 137 teeth with vertical preparations.^19^

Several limitations to this retrospective audit need to be considered. Treatment was provided by two clinicians at one practice. The follow-up was recorded by multiple clinicians and hygienists, as part of continuing care for these patients. There was no inter-examiner calibration between clinicians and hygienists. All data were gathered as part of continuing care for patients and there was no set data gathered for this audit. As such, not all data are available for all crowns. The crowns were not placed simultaneously. The same type of margin was prepared for all crowns, but different CAD/CAM systems were used. Some crowns were monolithic zirconia and some were zirconia veneered with ceramic, depending on the clinical circumstances. Only patients with follow-up data were included in the audit.

If the audit were to be run again, it would have been valuable to record the position of the crown margin at cementation and again at review visits. This would allow assessment of the role of gingival recession if present. It would be ideal if the review appointments could be carried out by an independent clinician.

This practice-based clinical data reflects treatment carried out in general practice. The nature of general practice means that individual cases need customised treatment which can impact the ability to compare results. On the other hand, general practice data tests the procedure in a real-world clinical setting.

Vertical margin preparation is technique-sensitive. Special care needs to be taken during removal of the crown bulbosity to ensure that no undercuts are left below the survey line. These undercuts cannot be blocked out by the technician, as might be possible with a horizontal margin, which may result in a crown margin which is not as apical as intended. Impression recording can be more challenging as there is no visible margin to inspect. Getting the margin on the provisional crown right is key to soft tissue management. It must be thin and well-contoured, which can be challenging with a temporary crown material. Determining the restoration margin position in the laboratory can be problematic but can be overcome by using polytetrafluoroethylene in the gingival sulcus to identify the position at the impression-recording stage.^20^ At try-in and cementation, care must be taken to seat the restoration slowly to allow excess cement to flow out. Working with a skilled technician who understands the concept is important in order to create a suitable margin and emergence profile.

The results from this audit and other reviews of vertical margin crowns are favourable and comparable with conventionally prepared crowns.^8^^,^^14^ Vertical preparations are more conservative of tooth structure - a key principle of restorative dentistry.

## Conclusions

With knife-edge margins now possible with zirconia, the vertical preparation is a credible option for crown placement or replacement. This enables greater conservation of natural tooth structure compared to preparations with horizontal margin designs. Tissue handling and margin form are critical to success.

This audit has shown favourable outcomes for 71 posterior teeth prepared with a vertical preparation for knife-edge zirconia crowns.

## Data Availability

Data can be made available subject to patient confidentiality requirements.
